# Identification of a novel *COL2A1* mutation (c.1744G>A) in a Japanese family: a case report

**DOI:** 10.1186/1752-1947-8-276

**Published:** 2014-08-14

**Authors:** Masaki Kishiya, Yoshihide Nakamura, Hirotaka Ohishi, Ken-Ichi Furukawa, Yasuyuki Ishibashi

**Affiliations:** 1 Department of Orthopaedic Surgery, Hirosaki University Graduate School of Medicine, 5 Zaifu-cho, Hirosaki, Aomori 036-8562, Japan; 2 Department of Pharmacology, Hirosaki University Graduate School of Medicine, 5 Zaifu-cho, Hirosaki, Aomori 036-8562, Japan

**Keywords:** *COL2A1* mutation, Avascular necrosis of the femoral head, Skeletal dysplasia

## Abstract

**Introduction:**

Mutations in the gene encoding the type II collagen gene (*COL2A1*) have been found to affect the entire skeletal system. Recently, inheritable skeletal dysplasia caused by novel *COL2A1* mutations has been linked to an inherited disease of the hip joint that neither involves the entire skeletal system nor is characterized by the presence of concomitant disorders, such as spinal or ocular abnormalities.

**Case presentation:**

A 27-year-old Japanese woman previously diagnosed with avasucular necrosis (AVN) of the femoral head on the basis of radiological findings was referred to the study site for surgical management of a painful hip joint. She had no history of disease but suffered from bilateral hip joint lesions. Analysis of her pedigree revealed that bilateral hip joint lesions affected more than three generations of her family. Based on these findings, haplotype analysis of her and her family members was performed by examining select candidate genes from the critical interval for epiphyseal dysplasia of the femoral head on 12q13 and sequencing the promoter and exonic regions of *COL2A1.*

**Conclusion:**

A novel *COL2A1* mutation (c.1744G>A) was identified within one Japanese family.

## Introduction

Mutations in the gene encoding the type II collagen gene (*COL2A1*) cause a series of type II collagenopathies that manifest as inheritable skeletal disorders, including achondrogenesis type 2 (ACG2; Langer-Saldino type), hypochondrogenesis, platyspondylic (Torrance type), congenital spondyloepiphyseal (SEDC), spondyloepimetaphyseal (SEMD) and Kniest dysplasia, Stickler syndrome type 1 and Stickler-like syndrome. These disorders often affect the entire skeletal system. Recently, such skeletal disorders have been reported to cause premature hip osteoarthritis (OA) [1], avascular necrosis (AVN) of the femoral head [2], and Legg-Calvé-Perthes disease (LCP) [3], and have been linked to an inherited disease of the hip joint that neither involves the entire skeletal system nor is characterized by the presence of concomitant disorders, such as spinal or ocular abnormalities.

## Case presentation

**Figure 1:**
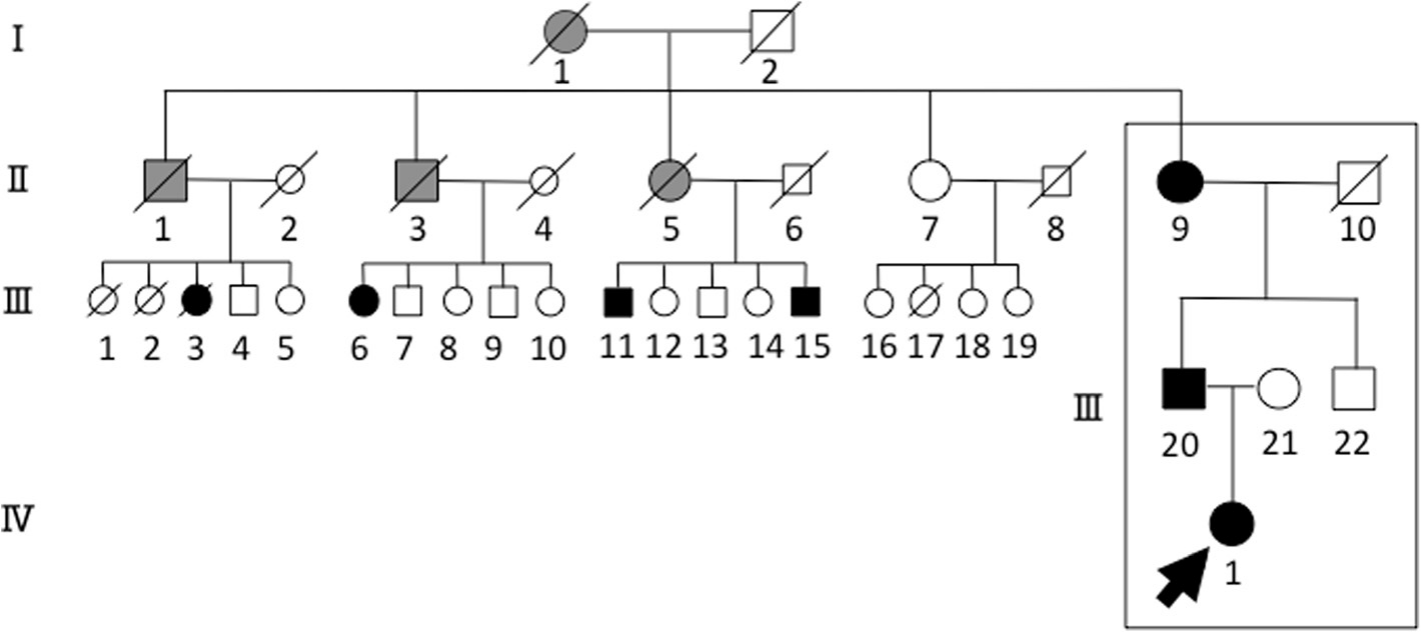
**Pedigree of our patient’s family.** Members I-1, II-1, 3, 5 (gray) had complained of coxalgia during their lifetimes and are suspected of having had hip joint disease. Members II-9, III-3, 20 had been diagnosed with end-stage OA and member III-3, 20 had required total hip arthroplasty. Members III-6, 11, 15 had been diagnosed with AVN and required trochanteric osteotomy. The proband (IV-1) is indicated by the black arrow.

**Figure 2:**
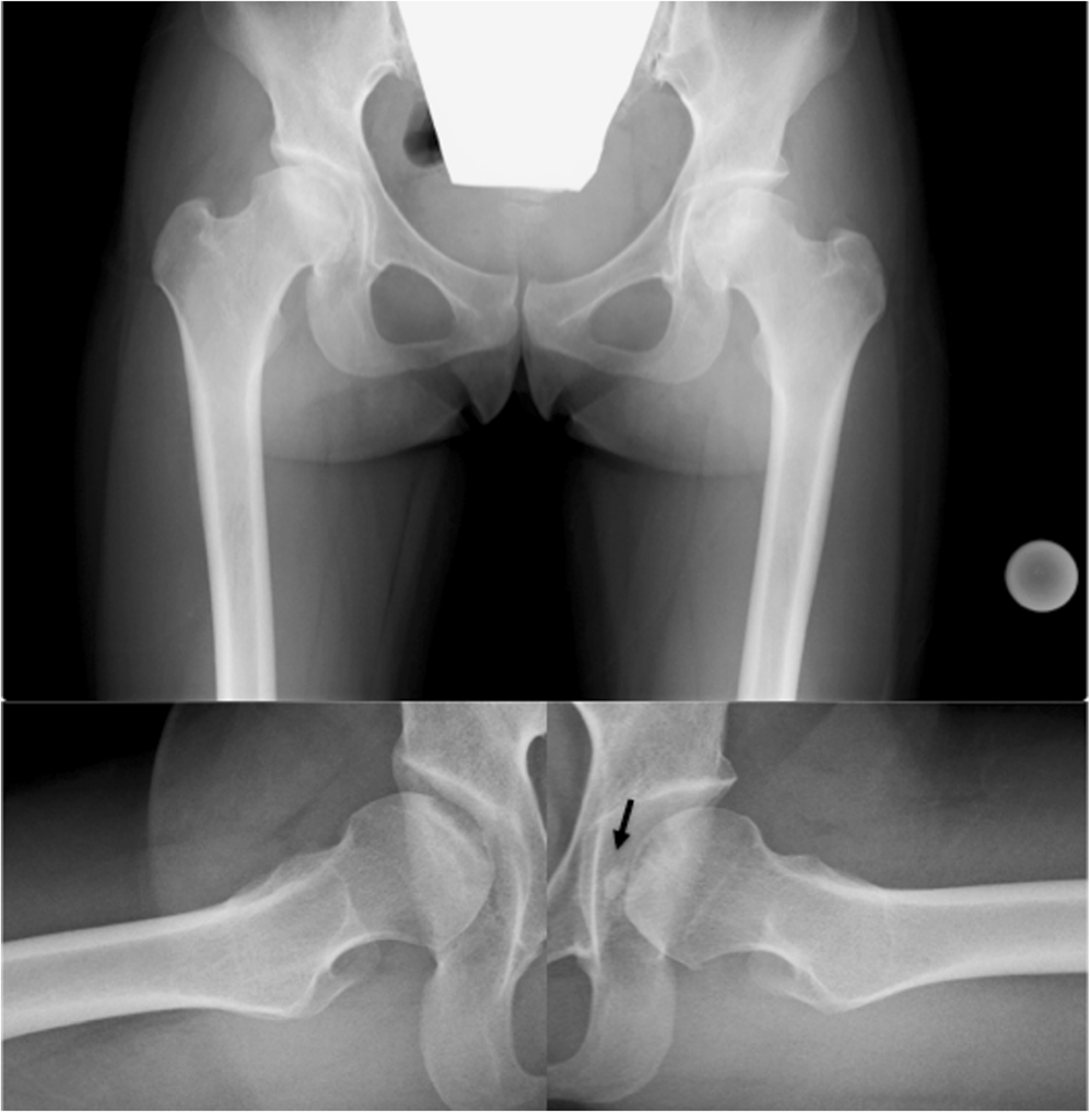
**Plain radiographic findings of the femoral head.** Anteroposterior (AP) and lateral radiographs (Sugioka) obtained at the time of the onset of pain show the crescent sign, collapse of femoral head, and no joint space narrowing in either femoral head. The lateral view (Sugioka) of left hip shows the presence of a free body (black arrow).

**Figure 3:**
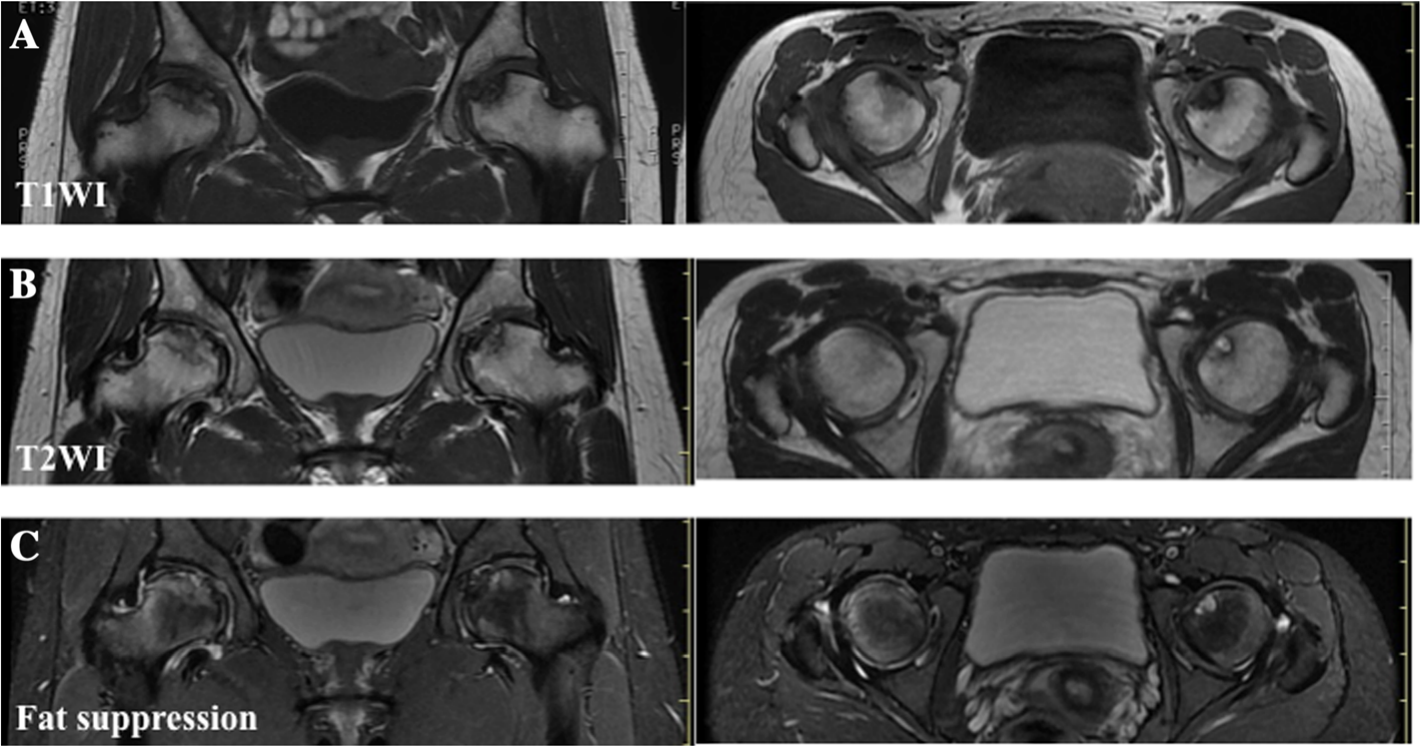
**Magnetic resonance imaging findings of the femoral head. A)** Coronal and axial slices of T1-weighted image show diffuse low-signal intensities in the femoral head. **B)** Coronal and axial slices of T2-weighted image show a combination of high- and low-signal intensities in the femoral head. **C)** This is a fat-suppression MRI in which part of the proximal portion beyond the low-intensity band shows contrast enhancement, an indication of perfusion.

**Figure 4:**
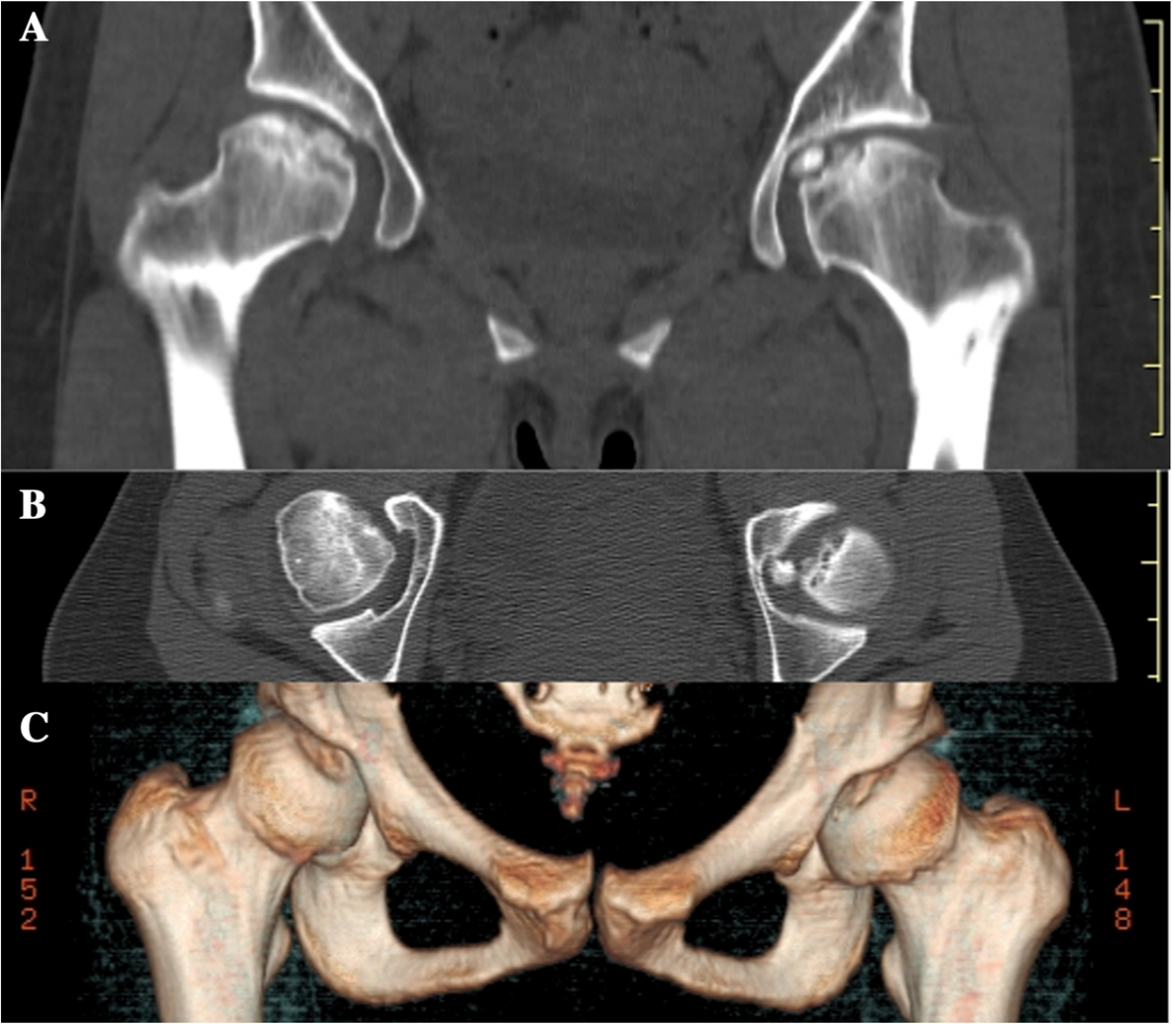
**Computed tomography findings of the femoral head.** Coronal **(A)** and axial **(B** and **C)** CT images of the right femoral head show a concave articular surface and a free body in the hip joint of the left femoral head. The free body is spherical and has a smooth surface.

**Figure 5:**
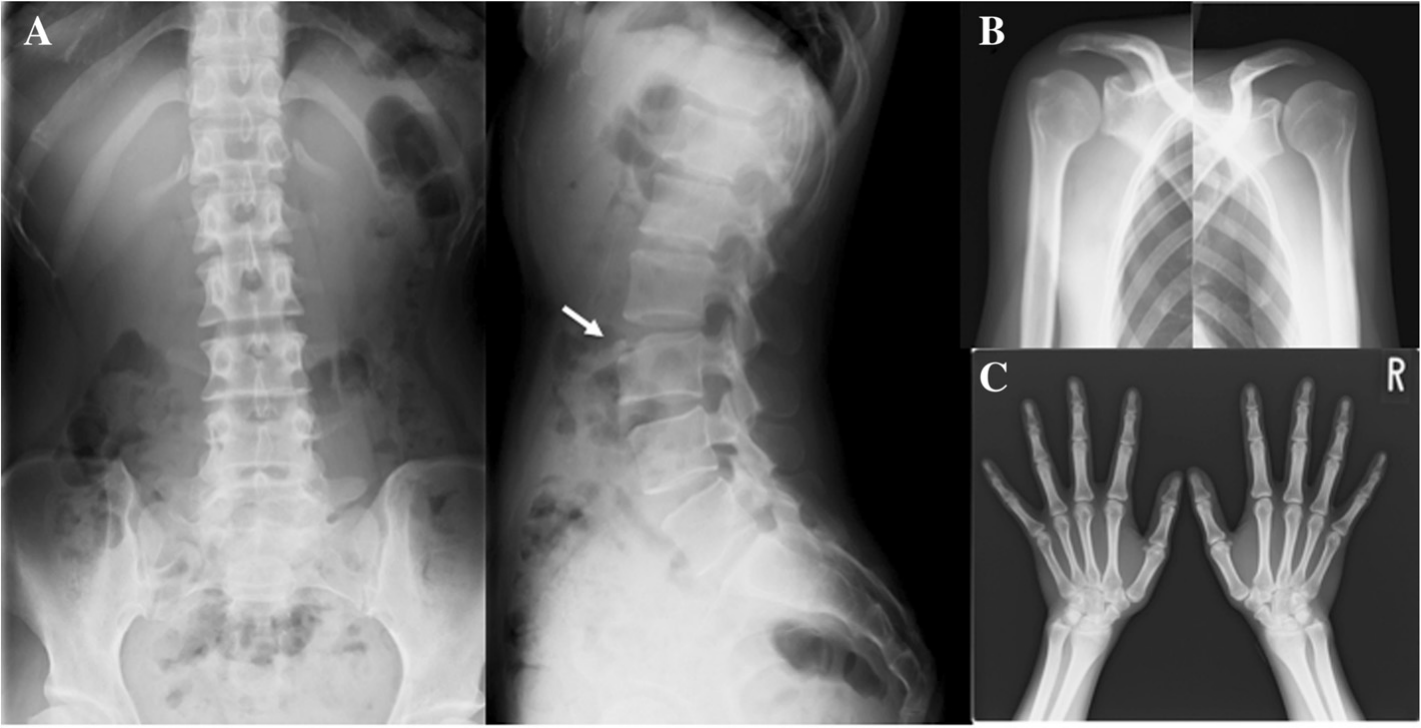
**Plain radiographic findings of the lumbar spine, shoulder and hand.** AP and lateral radiographs of the spine **(A)** show the third lumbar anterior goniodialysis (white arrow and circle) while AP radiographs of the shoulder **(B)** and hand **(C)** reveal almost normal findings.

**Figure 6:**
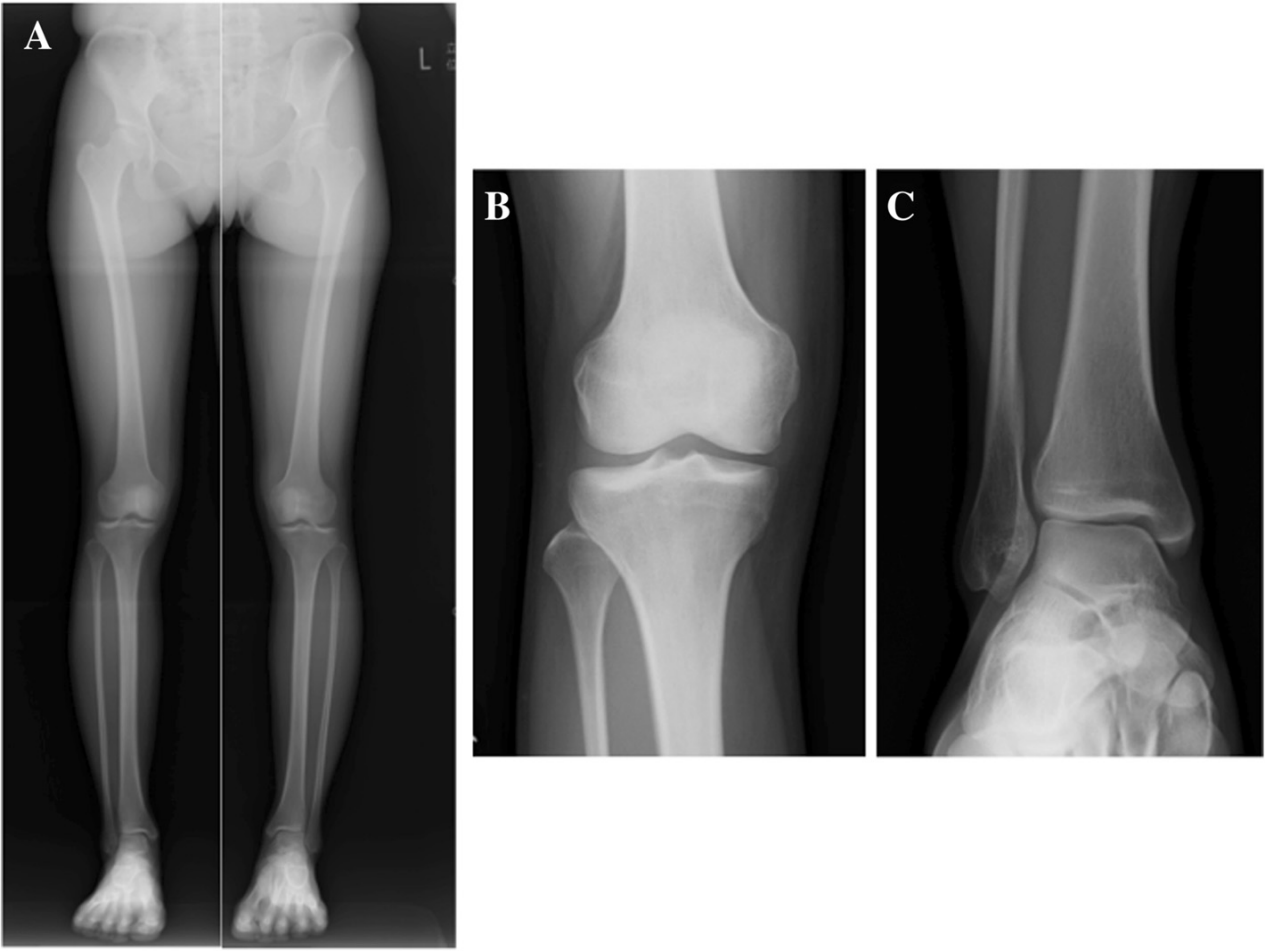
**Plain radiographic findings of the full length of the lower limbs, knee and ankle.** AP radiographs of the full length of the lower limbs **(A)**, knee **(B)**, and ankle **(C)** reveal almost normal findings with no limb shortening.

A 27-year-old Japanese woman was admitted to a local hospital for treatment of a painful bilateral hip joint. Based on assessment of the radiological findings, she was diagnosed with AVN and referred to the study site for surgical management of her condition. The patient had no history of disease (for example, systemic lupus erythematosus (SLE) or human immunodeficiency virus (HIV)), alcohol abuse, steroid use, trauma or diving (causing dysbarism), and her laboratory findings were normal. However, as shown in her family pedigree, and her family clinical findings, more than three generations of her family have hip joint lesions: the proband (IV-1), her father (III-20) and her grandmother (II-9), II-1, 3, 5, III-3, 6, 11, 15 (Figure 1, Table 1). These findings suggest the presence of autosomal dominant inheritance with a high level of penetrance within this family (not shown).Although our patient’s radiological findings indicated osteonecrosis-like lesions of both femoral heads, the plain radiographic (Figure 2), magnetic resonance imaging (MRI) (Figure 3), and computed tomography (CT) (Figure 4) findings were negative for AVN induced by steroids and alcohol. Our patient’s anteroposterior (AP) radiographs of the other sites (knees, ankles, spine, hands, elbows) revealed almost normal findings (Figures 5 and 6).

**Table 1:** Clinical findings of patient and family with lesions of the femoral head

	**Sex**	**Height (cm)**	**Body weight (Kg)**	**BMI**	**Diagnosis**	**Treatment**
**Grandmother**	Female	135	35	19.2	Osteoarthritis	conservative treatment
**(II-9)**						
**Father**	Male	156	62	25.5	Osteoarthritis	THA
**(III-20)**						
**Pedigree**	Female	149	39	17.6	Skeletal dysplasia	Trochanteric curved varus osteotomy
**(IV-1)**						
**II-3**	Female	150	50	22.2	Osteoarthritis	THA
**III-6**	Female	143	42	20.6	ION	Trochanteric osteotomy
**III-11**	Male	150	50	22.2	ION	Trochanteric osteotomy
**III-15**	Male	158	67	26.8	ION	Trochanteric osteotomy

Table 1 shows the clinical findings of more than three generations of the patient’s family who have hip joint lesions: the proband (IV-1), her father (III-20) and her grandmother (II-9), II-1, 3, 5, III-3, 6, 11, 15. *1 BMI, Body mass index; *2 THA, Total hip arthroplasty; *3 AVN, Avascular necrosis.

**Figure 7:**
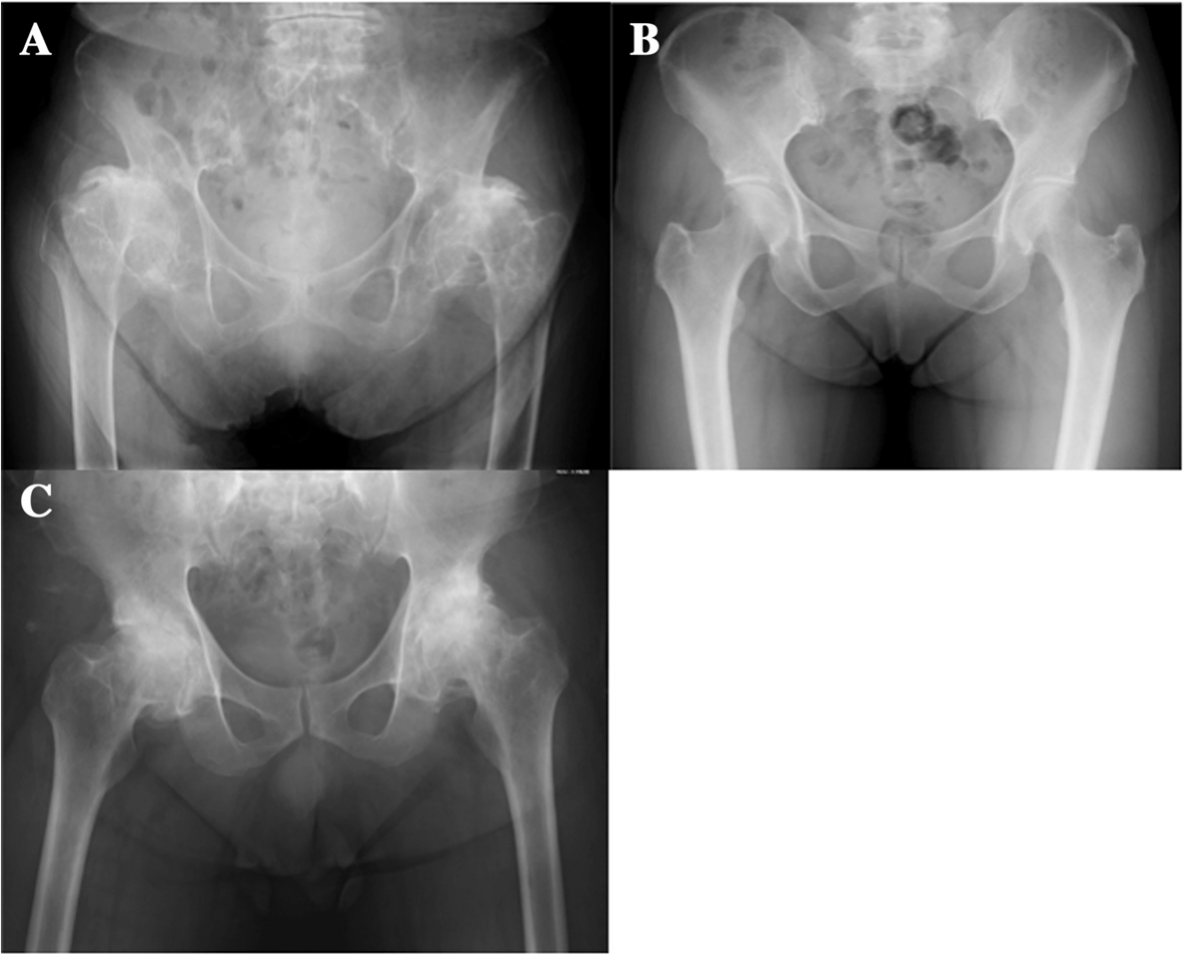
**Plain radiologic findings of our patient’s family.** Plain radiographs of our patient’s grandmother **(A)** and father **(B)** show end-stage OA, while a radiograph of our patient’s mother **(C)** appears almost normal.

**Figure 8:**
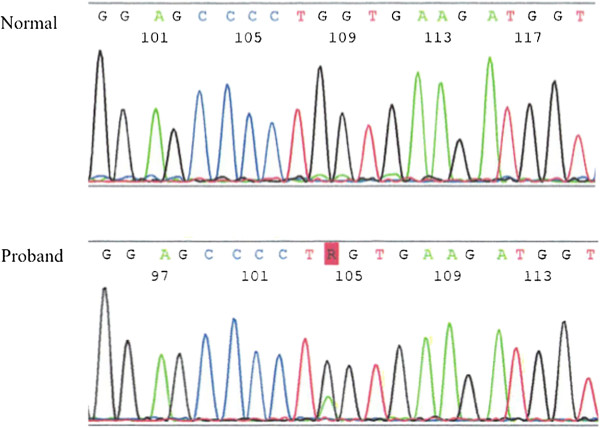
***COL2A1*mutations in our patient.** Sequencing analysis shows a G→A transition of *COL2A1* (c.1744G>A (p.Gly582Ser)). This transition predicts the replacement of glycine with serine at codon 382 in *COL2A1*.

**Figure 9:**
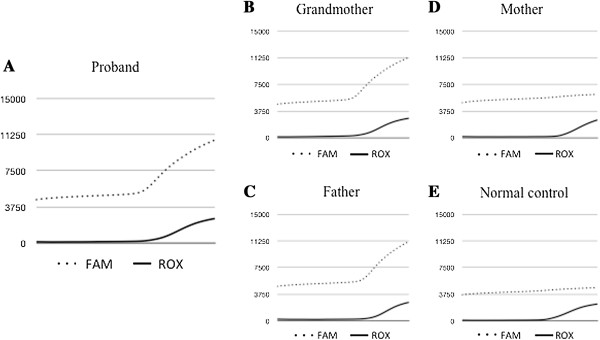
**Real-time polymerase chain reaction analysis of our patient’s family and a normal control.** Probe-adapted real-time PCR analysis using cycling probe technology led to the detection of a single nucleic acid polymorphism (SNP) in a target DNA sequence in our patient’s father and grandmother. For SNP typing, two cycling probes labeled with two different fluorescent dyes (6-carboxyfluorescein (FAM) or 6-carboxy-X-rhodamine (ROX)) were used, with each probe harboring ribonucleic acid (RNA) corresponding to the wild-type nucleotide or the nucleotide with a mutation at the SNP position. Expression analysis of our patient **(A)**, her father **(B)** and her grandmother **(C)** shows a G→A transition of *COL2A1* (c.1744G>A), while expression analysis of her mother **(D)** and a normal control **(E)** shows no mutation in the *COL2A1* coding region.

Haplotype analysis of the family members was performed by examining select candidate genes from the critical interval for epiphyseal dysplasia of the femoral head on 12q13. The entire coding regions of *COL2A1*, along with the flanking intronic regions, were amplified by polymerase chain reaction (PCR) using the Ex Taq™ system with an ABI PRISM® 3100 Genetic Analyzer (Applied Biosystems, Foster, CA, USA) and a BigDye® Terminator Cycle Sequencing Kit (Applied Biosystems). The complementary deoxyribonucleic acid (cDNA) sequence of *COL2A1* was obtained from GenBank (accession no. NM_001844.4). For cDNA numbering, +1 corresponds to the A of the ATG translation initiation codon 1 in the reference sequence (Figure 7). A G→A transition of *COL2A1* (c.1744G>A (p.Gly582Ser)) was detected in the affected member in each of the three generations of the family (Figures 8 and 9). This transition predicts the replacement of glycine with serine in *COL2A1*. Based on this finding, members of the family were diagnosed with inherited epiphyseal dysplasia located on the femoral head. For those three affected members in whom autosomal dominant inheritance of the disease was strongly suspected (IV-1, III-20, II-9), the chromosomal position of the gene to *COL2A1* (c.1744G>A (p.Gly582Ser)) was mapped. No mutation was detected in the *COL2A1* coding region of the mother or in a normal control (Figure 9).

## Discussion

In this study, we identified a novel *COL2A1* mutation (c.1744G>A (p.Gly582Ser)) from the chromosome 12q13 region within one Japanese family. A history of hip disorder and typical radiologic findings lend support to the idea that the identified mutation in the *COL2A1* gene transition might be the cause of the disease in this family.

*COL2A1* mutations typically manifest as inheritable skeletal disorders often affecting the entire skeletal system [4]. Except for the hip joint, the affected members of this family were otherwise normal, with normal spine development. Moreover, they had no anomalies of the ocular or auditory system.

Recently, inheritable epiphyseal dysplasia caused by novel *COL2A1* mutations has been linked to three inherited diseases of the hip joint. Kannu *et al.* reported premature osteoarthritis as well as LCP as presenting with *COL2A1* mutation [1,5]. On the other hand, Liu *et al.* reported three families with AVN which were carriers of the *COL2A1* mutation [2]. Recently, Su *et al.* reported that age at onset in relation to closure of the femoral head epiphysis is a critical factor in determining the disease pattern [6]. However, there is no clear consensus as to inherited hip disease induced by *COL2A1* mutations. In this study, the father of our patient who, along with her grandmother, had been diagnosed with end-stage OA, had required total hip arthroplasty, while several of her more distant relatives diagnosed with AVN had required trochanteric osteotomy and total hip arthroplasty.

Osteoarthritis, AVN and LCP are difficult to distinguish, as both symptoms and clinical findings are similar. We believe that we should recognize familial hip dysplasia induced by *COL2A1* mutation. However, the diagnosis will differ depending on the age at presentation to hospital. It is predicted that cartilaginous structure abnormality is already in existence at the time of the growth spurt. In addition, in joint cartilage, type 2 collagen forms a complex with proteoglycan. A decrease in this type II proteoglycan complex results in a decrease in cartilaginous elasticity. When there is disease progression without symptoms, cartilaginous abrasion and damage and subchondral bone exposure occurs until, ultimately, severe OA results. Nishimura *et al.* reported that glycine to serine substitutions resulted in alternating zones that produce severe and milder skeletal phenotype [4]. Our result was similar, as this transition predicted the replacement of glycine with serine in *COL2A1* (p.Gly582Ser). The patient, whose lesions were localized to the hip joint, had neither a remarkable clinical history nor remarkable radiological (Computed radiography (CR), MRI, and CT) characteristics. As her family findings indicated, the phenotypic spectrum of *COL2A1* mutations may be difficult to diagnose, leading to concern regarding the possibility of misdiagnosis as type II collagenopathy presenting as LCP [3] (in patients 0 to 18 years of age), AVN [2] (in patients 18 to 45 years of age) or OA (in patients 45 years of age and older). The findings of this study also indicate that the severity of the disease might be affected by the site of the *COL2A1* mutation.

We believe that new discoveries in molecular genetics have allowed for identification of the genes causing a large number of skeletal disorders, improving the means of their classification and differentiation.

## Conclusion

A novel *COL2A1* mutation (c.1744G>A (p.Gly582Ser)) was identified within one Japanese family with epiphyseal dysplasia. Genetic analysis revealed that more than three generations carried the *COL2A1* mutation causing this disorder.

## Consent

This study was approved by the Ethics Committee of Hirosaki University Graduate School of Medicine and conducted according to the principles of the Declaration of Helsinki. Written informed consent was obtained from our patient for publication of this case report and any accompanying images. A copy of the written consent is available for review by the Editor-in-Chief of this journal.

## Abbreviations

ACG2: Achondrogenesis type 2; AVN: Avascular necrosis; *COL2A1*: Collagen, type II, alpha 1; HIV: Human lummunodeficiency virus; LCP: Legg-Calvé-Perthes disease; OA: Osteoarthritis; SEDC: Spondyloepiphyseal dysplasia congenita; SEMD: Spondyloepimetaphyseal dysplasia; SLE: Systemic lupus erythematosus; SNP: Single nucleic acid polymorphism.

## Competing interests

The authors declare that they have no competing interests.

## Authors’ contributions

MK analyzed and interpreted the patient data regarding the skeletal dysplasia. YN, HO and KF were involved in reviewing the literature and proofreading the manuscript, and were major contributors in writing the manuscript. YN performed the final revisions of the manuscript. YI is the senior author and was responsible for final proofreading of the article. All authors read and approved the final manuscript.
